# Zidovudine/Lamivudine vs. Abacavir/Lamivudine vs. Tenofovir/Emtricitabine in fixed-dose combinations as initial treatment for HIV patients: a systematic review and network meta-analysis

**Published:** 2017-06-30

**Authors:** Juan Carlos Alzate Angel, Marcela María Duque Molina, Héctor Iván García García

**Affiliations:** 1 Corporación para Investigaciones Biológicas, CIB (Corporation for Biological Research), Medellín, Colombia; 2 Academic Group of Epidemiology, Faculty of Medicine, Universidad de Antioquia, Medellín, Colombia; 3 Asistencia Científica de Alta Complejidad (Highly Complex Scientific Assistance), Medellín, Colombia

**Keywords:** HIV, emtricitabine, tenofovir disoproxil fumarate drug combination, abacavir, lamivudine drug combination, lamivudine, zidovudine drug combination, systematic review, network meta-analysis

## Abstract

**Introduction:**

Initial treatment of the HIV is based on the use of three drugs, two of which are nucleoside analog reverse-transcriptase inhibitors. There are three combinations of these drugs which have been approved by different guidelines, each with divergent results in terms of efficacy and safety.

**Objective:**

To compare the efficacy and safety of these three combinations.

**Methods:**

Systematic review and network meta-analysis of randomized clinical trials comparing fixed doses of Tenofovir Disoproxil Fumarate / Emtricitabine (TDF/FTC), Abacavir / Lamivudine (ABC/3TC) and Zidovudine / Lamivudine (ZDV/3TC).

**Results:**

Seven clinical trials met the eligibility criteria. The results suggested higher efficacy with TDF/FTC vs. ABC/3TC at 96 weeks and vs. ZDV/3TC at 48 weeks. However, there is clinical and statistical heterogeneity. Subgroup analysis were performed by third drug and by level of viral load prior to treatment, and found no differences in virological control. Network meta-analysis could only be carried out with TDF/FTC vs. ZDV/3TC, and the proportion of patients with virological response, with no differences at 48 weeks nor at 96 weeks. Direct comparisons showed an increased risk of bone marrow suppression of ZDV/3TC vs. TDF/FTC and of ABC/3TC hypersensitivity reactions vs. ZDV/3TC

**Conclusions:**

The results did not show differences in effectiveness among the interventions. However, due to the heterogeneity of the third drug and the follow-up time between the included studies, this result is not definitive. The results raise the need for further studies to help improve treatment recommendations in patients infected with HIV.

## Resumen

**Introducción:**

El tratamiento inicial de la infección por VIH se basa en el uso de tres medicamentos, dos de ellos inhibidores de transcriptasa reversa análogos de nucleósido. Existen tres combinaciones de estos medicamentos aprobadas por diferentes guías, con resultados divergentes en cuanto a eficacia y seguridad.

**Objetivo:**

Comparar la eficacia y seguridad de las 3 combinaciones

**Métodos:**

Revisión sistemática y metanálisis en red de ensayos clínicos con asignación aleatoria comparando dosis fijas de Tenofovir Disoproxil Fumarato/Emtricitabina (TDF/FTC), Abacavir/Lamivudina (ABC/3TC) y Zidovudina/Lamivudina (ZDV/3TC).

**Resultados:**

Siete ensayos clínicos cumplieron los criterios de elegibilidad. Los resultados sugirieron mayor eficacia con TDF/FTC vs ABC/3TC a 96 semanas y vs. ZDV/3TC a 48 semanas. Sin embargo, existe heterogeneidad clínica y estadística. Se realizó análisis de subgrupos por tercer medicamento y por nivel de carga viral previa al tratamiento, sin encontrar diferencias en control virológico. Se pudo realizar metanálisis en red con TDF/FTC vs ZDV/3TC y proporción de pacientes con respuesta virológica, sin diferencias a las 48 semanas ni 96 semanas. Las comparaciones directas evidenciaron mayor riesgo de supresión de médula ósea de ZDV/3TC vs TDF/FTC y de reacciones de hipersensibilidad de ABC/3TC vs ZDV/3TC.

**Conclusión:**

Los resultados no demostraron diferencias en efectividad entre las intervenciones; sin embargo, debido a heterogeneidad en cuanto al tercer medicamento y el tiempo de seguimiento entre los estudios incluidos, dicho resultado no es definitivo. Los resultados plantean la necesidad de realizar nuevos estudios que ayuden a mejorar las recomendaciones de tratamiento en los pacientes infectados por el VIH.

## Introduction

It has been more than 30 years since five young homosexual men were reported to have a rare *Pneumocystis carinii* pneumonia disease at three hospitals in Los Angeles, USA ^1^. Several events occurred until the definition of Acquired Immunodeficiency Syndrome (AIDS) in 1982 came about ^2^. It was not long before the few initial cases became tens of millions, generating one of the greatest pandemics of modern times ^3^.

Twenty-six drugs, which reduce mortality caused by the human immunodeficiency virus (HIV), have been approved for the treatment of HIV. The reduction in initial costs of antiretroviral drugs, the availability of generic drugs and the increase in international financing have marked the expansion of its use in low- and middle-income countries ^3^.

Different societies with different health systems have issued antiretroviral treatment guidelines for adults and adolescents, with periodic updating of their recommendations. The guidelines of the European AIDS Clinical Society (EACS), the World Health Organization (WHO) and the International Antiviral Society (USA) define recommendations for preferred treatment schedules for the initiation of antiretroviral therapy, with some differences between them: the WHO guidelines clarify that in cases where tenofovir disoproxil fumarate/emtricitabine (TDF/FTC) cannot be used, zidovudine/lamivudine (ZDV/3TC) may be used and that schedules containing abacavir (ABC) are not considered preferred alternatives. The European Guidelines establish, as preferred treatment schedules, those containing TDF/FTC or abacavir/lamivudine (ABC/3TC), clarifying that the latter should be used with caution in cases with viral loads greater than 100,000 copies/mL. Finally, the USA Panel recommends several different initial schedules, each with an indication or a warning about its use depending on baseline viral load, the third drug chosen in the schedule, and patient-specific conditions that contraindicate the use of one or more of the available medications, making it clear that most of the schedules contain TDF/FTC or ABC/3TC as a treatment cornerstone ^4^ - ^6^.

In Colombia, patient care has been based on the *Guía para el manejo del VIH/SIDA. Basada en la evidencia* (Guidelines to Managing HIV/AIDS. Evidence-based). In it zidovudine (ZDV) plus lamivudine (3TC) were recommended as the preferred nucleoside reverse-transcriptase inhibitors ^7^ ^,^ ^8^. In the 2014 update of this guideline, the ZDV/3TC schedule became recommended as an alternative treatment schedule ^9^. In other Latin American countries, such as Argentina and Chile, the three combinations remain as the schedules recommended when initiating therapy ^10^ ^,^ ^11^.

All this makes it clear that worldwide, there are two preferred nucleoside reverse-transcriptase inhibitor schedules to be included in antiretroviral therapy (ART) and that in some countries, the ZDV/3TC option is still recommended or considered as an alternative.

The results of direct comparisons of the efficacy and safety of the aforementioned treatment schedules have shown similar effectiveness between schedules containing TDF/FTC vs. ZDV/3TC, with differences in terms of the safety of schedules, even when they are differentiated by gender ^12^ ^,^ ^13^. Other studies have found that medications such as 3TC and FTC are clinically equivalent ^14^. In some studies comparisons of combinations of ABC/3TC vs. TDF/FTC showed similar antiviral efficacy ^15^ ^,^ ^16^, while in another study, greater risk of virologic failure was found in those using ABC/3TC when viral load was greater than 100,000 copies/mL ^17^.

As for the safety of the different treatment schedules, there are also divergent results. Differences have been reported in terms of renal side effects, with increased markers of tubular dysfunction in patients receiving TDF/FTC ^18^ ^,^ ^19^, without clarity on the clinical relevance of such findings. A greater impact on bone density was observed with TDF/FTC based treatment schedules compared to ABC/3TC based schedules ^20^ ^,^ ^21^.

There are no comparisons that include these three schedules, so the aim of this research was to compare the efficacy and safety of the combination of ZDV/3TC vs. ABC/3TC vs. TDF/FTC as components of highly active antiretroviral therapy (HAART) in patients more than 18 years old with HIV who are initiating treatment.

Systematic reviews of randomized clinical trials are considered the standard in evidence-based health care decisions and many systematic reviews use meta-analysis to combine quantitative results and summarize available evidence. Meta-analysis can improve knowledge about a therapeutic strategy by increasing statistical potency and precision in the size of the treatment effect, or by resolving controversies that arise from seemingly contradictory studies. However, they may have limitations that could affect the validity of the results obtained, as is the case with combining studies that have different clinical characteristics among the participants. They can also produce erroneous results if studies are combined that have a risk of bias, and thus generate an inappropriate overview. These limitations must be taken into account prior to performing meta-analysis in order to adequately manage these limitations that then lead to obtaining valid and generalizable results ^22^. Ideally clinical trials should simultaneously compare all interventions of interest, however, such studies are almost never available. In the absence of studies involving a direct comparison, an indirect comparison can provide useful evidence. Similarly, the combination of direct and indirect evidence may strengthen the evaluation of available interventions ^23^.

## Materials and Methods

A systematic review and network meta-analysis were conducted, which included parallel randomized trials, undertaken for any purpose (equivalence, superiority, noninferiority), in a single center or multicenter, in any language, in any country and with any follow-up time. We included studies conducted with patients over 13 years of age, with a confirmed diagnosis of HIV through any direct or indirect confirmatory test, where it was decided to start HAART and who were not previously exposed directly to any antiretroviral drug.

The interventions that were compared were TDF/FTC co-formulations (300/200 mg orally every 24 h), ABC/3TC (600/300 mg orally every 24 h) and ZDV/3TC (300/150 mg orally every 12 h) that would have been performed in direct comparison with each other or with a placebo. 

### Outcome measures 

The outcomes were: (i) mortality; (ii) clinical progression to AIDS (proportion of patients who in the studies have a defined AIDS disease or progress to stage C and/or stage 3 classification of the Centers for Disease Control and Prevention (Atlanta, USA) of 1993 or 2008, after initiating antiretroviral therapy ^24^ ^,^ ^25^; (iii) virological response to antiretroviral therapy defined as the proportion of patients achieving a viral load below 50 copies/mL at 48 and 96 weeks after initiating antiretroviral therapy ^6^; (iv) virological failure (HIV viral load >50 copies/mL 6 months after initiating therapy in people who continue with antiretroviral therapy) ^6^; (v) adherence to treatment (proportion of patients who, at the end of the study, continue with the same initial treatment schedule without interruptions); (vi) immunological failure (CD4 count falling from baseline or persistently less than 100 cells/μL) ^5^; (vii) hypersensitivity reaction to ABC (multiple organ syndrome occurring within the first 6 weeks after initiating ABC treatment) ^4^; (viii) proportion of new cases of Acute Myocardial Infarction (AMI) or Acute Cerebrovascular Disease (CVD) after initiating antiretroviral therapy; ix) bone marrow suppression defined as the proportion of new cases of anemia and/or neutropenia after initiating antiretroviral therapy; x) lactic acidosis (increase of serum lactate >5 mmol/L associated with systemic symptoms) ^6^; (xi) lipodystrophy (increased or decreased subcutaneous fat measured by anthropometry or Dual-energy X-ray absorptiometry - DXA) ^26^; (xii) renal abnormalities (tubulopathies, nephrolithiasis, interstitial nephritis) ^6^; and (xiii) osteopenia (postmenopausal woman or man aged ≥50 years with bone mineral density measured by the DXA T-score -1 to -2.5) osteoporosis (postmenopausal woman or man with ≥50 years with bone mineral density measured by the DXA T-score ≤-2.5 or premenopausal woman or man with <50 years with bone mineral density measured by DXA Z-score ≤-2 and fragility fractures) ^6^.

### Search methods to identify studies

All searches were conducted without language or country restriction. They were limited to studies on humans, from 1995 (the beginning of HAART) until May 2014.

An electronic search was conducted in the following databases: MEDLINE, EMBASE, Cochrane Central Register of Controlled Trials (CENTRAL), Cochrane Database of Systematic Reviews (CDSR), Latin American and Caribbean Health Sciences Literature (LILACS), African Index Medicus (AIM), International Clinical Trials Registry Platform (WHO), ClinicalTrials. Furthermore, abstracts, posters, talks given at conferences on Retroviruses and Opportunistic Infections (CROI), international HIV conferences, AIDS Clinical Trials Group Network (ACTG) were searched. Searches were conducted in the following journals: New England Journal of Medicine, Journal of the American Medical Association (JAMA), the Lancet, and the Journal of the International AIDS Society. We reviewed the references of all studies found.

The search terms used were "abacavir", "lamivudine", "tenofovir", "emtricitabine", "zidovudine", "randomized controlled trial", "controlled clinical trial", "randomized", "placebo" drug therapy", "randomly", "trial", "groups" (Appendix 1).

### Selection of studies

The final selection of studies was done by two independent reviewers, both experts in the care of patients with HIV in Colombia and students of epidemiology, with advice from a librarian, expert in health sciences database searches.

Both reviewers assessed all titles and abstracts and excluded those considered irrelevant to the review as they did not meet the inclusion criteria or were duplicates. Subsequently, they evaluated the complete written text of each study to verify the eligibility criteria. Agreement between the two reviewers was assessed using simple kappa statistics, resolving disagreements through discussion between the two.

### Selection and handling of variables

Variables -belonging to the following types that were considered relevant for the comparison of the studies and for the measurement of outcomes- were selected: source; eligibility; methods; participants; interventions; outcomes; results; and funding source.

### Assessment of risk of bias in studies included

We performed the criteria recommended by Cochrane Collaboration ^22^ and the Review Manager 5.3 program, which included a review of the random sequence generation, allocation concealment, blinding of participants and personnel, blinding of the outcome assessment, reporting and management of lost data, selective reporting and other potential biases.

### Geometry of the network

The geometry of the network was defined according to the direct comparisons found in the studies chosen after meeting the eligibility criteria and a review by the evaluators. If among all the included studies there was at least one direct comparison between each of the evaluated treatments, it was defined that the geometry of the network corresponded to a closed loop ^23^.

### Measures of treatment effect

After analyzing the type of outcomes studied which correspond to proportions (number of patients with HIV compared to the total number of patients assigned to each treatment) and analyzing the advantages and disadvantages of each measure of the effect -taking into account that relative measures are more consistent than absolute measures as is the ease of interpretation by clinicians ^22^, it was defined that the most consistent measure with our outcomes is relative risk, with a respective 95% confidence interval. When the data were extracted, when a reported outcome with continuous measures was found, it was analyzed whether it was possible to extract the means and the standard deviations in order to analyze by means difference.

### Analysis methods

#### Synthesis of data

The estimation of the effect of each outcome was performed initially by meta-analysis of direct comparisons in order to obtain the effect of each combination and to be able to use it later in the network meta-analysis. To that effect, considering that the studies could have reported a small number of occurrences and that the generic method of inverse variance may be less robust in this context, the Mantel Hanzel method was used for dichotomous results, with relative risk as a measure of effect. Similarly, the comparisons were analyzed by random effects analysis, because due to conditions such as variability of the third drug between studies, it cannot be concluded that there is no variation between the size of the effect between studies. In each measurement, clinical heterogeneity was assessed by investigating and comparing the baseline characteristics of the participants included in each study (comparative interventions, age, gender, viral load, CD4+ T lymphocytes count), and heterogeneity (variability in the effects of the intervention) using the statistic I². When clinical or statistical heterogeneity (I² greater than 40%) was found, the variables considered as effect modifiers -defined as those characteristics of the patients or the studies that may be associated with the final effect of the treatment ^27^- were evaluated by means of subgroup analysis and if these variables were homogeneous among the comparisons evaluated, a statistical analysis by means of meta-analysis was determined ^22^.

The total number of patients exposed in each outcome corresponded to the total number of patients randomly assigned to each comparison, since the analyzes in each study were by intention to treat and in order to preserve the random assignment as an assumption for direct and indirect comparisons.

#### Indirect comparisons

According to the geometry of the network found and having a priori the possibility that it was closed loop, with a minimum direct comparison available between two interventions, it was put forward that a final analysis for each outcome be performed using the method of mixed comparisons, or Bucher's method of indirect comparisons. These methods have as a fundamental assumption that comparisons occur through a closed loop for mixed comparisons and that the relative efficacy of one treatment is the same in all studies included in the indirect comparison for the Bucher method. Additionally, for their results to be valid, the effect of any treatment must be interchangeable through the other studies in the network. This method has the strength to preserve random assignment ^28^.

There are several statistical programs that allow comparisons to be made, and in the case of comparisons with networks, such as ours with three interventions, where the individual studies are comparisons of two branches, simple methods have been designed in Excel sheets for obtaining the results ^29^.

#### Evaluation of inconsistency

In order to carry out indirect comparisons, it is essential that the principles of transitivity be observed, that is, the similarity between the variables modifying the effect and the consistency between the studies, defined as the agreement between the direct and indirect sources of evidence and that it be evaluated statistically by the inconsistency factor through the specific loop approximation.

#### Evaluation of reporting biases

To evaluate the presence of bias the following strategies were used: funnel plot and prevention of language bias, duplicate publication, location, and citation by strict compliance with the methodology designed for the systematic review.

#### Subgroup analysis

According to previous knowledge and reports on studies, the main causes of heterogeneity in the effects of each treatment, also defined as effect modifiers, were considered to be the viral load level and the third drug. For this reason, subgroup analysis was performed according to baseline viral load prior to initiating treatment, assessing differences in effects between subgroups with viral loads greater than or equal to 100,000 copies/mL and less than 100,000 copies/mL and the differential response according to the third drug (non-nucleoside reverse-transcriptase inhibitor, protease inhibitor or integrase inhibitor).

## Results

### Selection of studies

**Figure 1:**
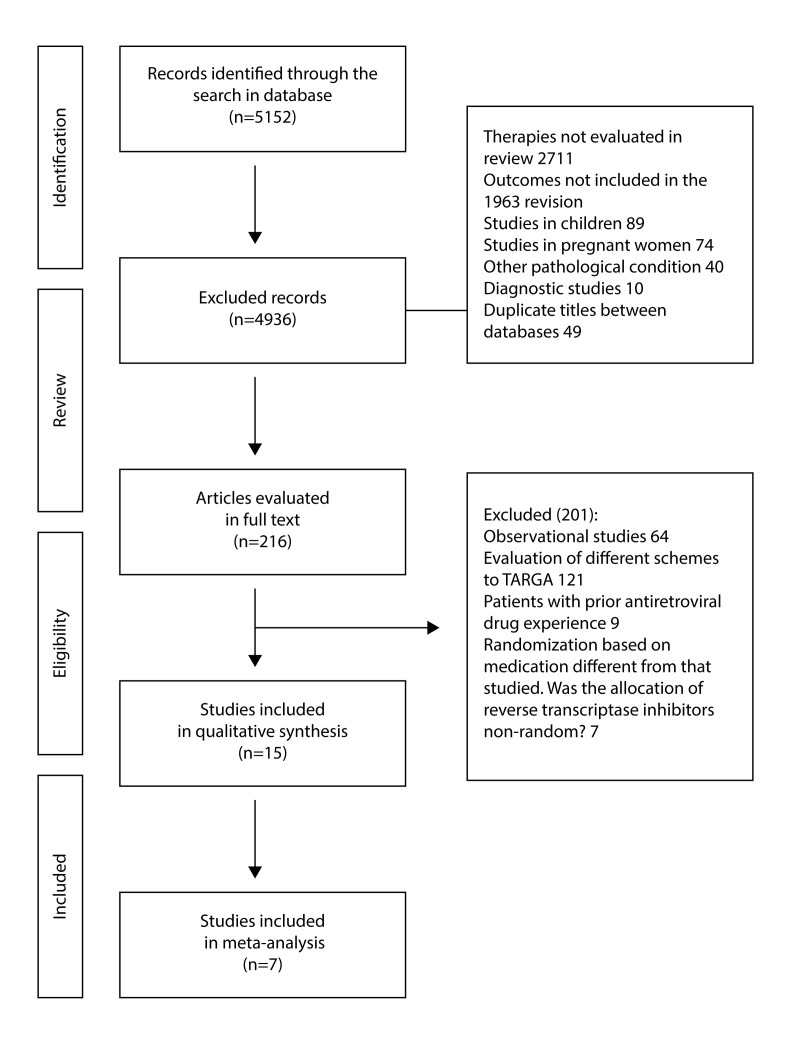
Flowchart with search results.

The search identified 5,152 titles from the initial evaluation, of which 4,936 were excluded after the revision of the title, abstract and due to possible duplication. 2,711 were excluded because the therapy evaluated did not correspond to the one included in the review, 1,963 evaluated outcomes not studied in our review, 89 were studies of children, 74 of pregnant women, 40 studied treatments for another pathological condition associated with HIV, 10 corresponded to diagnostic studies and 49 duplicate titles were detected between the databases. The remaining 216 articles were reviewed fully, verifying eligibility criteria. Following this review, we obtained 15 publications corresponding to 7 studies, 5 comparing ABC/3TC vs. TDF/FTC, one ZDV/3TC vs. TDF/FTC and one ABC/3TC vs. ZDV/3TC (Fig. 1).

The agreement between the two reviewers was evaluated using a simple kappa statistic, obtaining a kappa of 0.9239 (CI 95%: 0.81-1.00). The two disagreements between the reviewers were resolved by discussion between them and there was no need to go to a third reviewer.

### Geometry of the network

**Figure 2:**
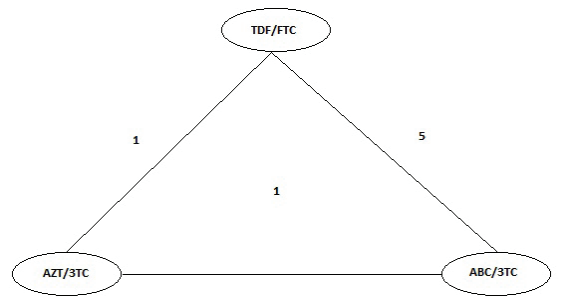
Evidence Network for Meta-analysis. The numbers between the nodes are equivalent to the number of studies that directly compared the interventions joined by the line.

After defining the studies included in the meta-analysis, the following network of direct comparisons was obtained, the number indicates the number of studies between each node (Fig. 2).

### Evaluation of risk of bias in studies included

**Figure 3:**
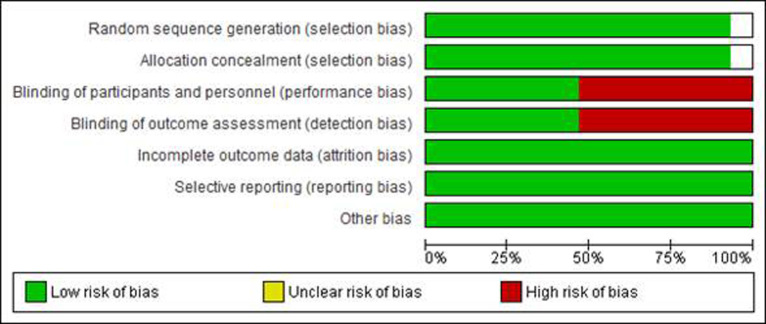
The Risk of Bias in the Clinical Trials Included in the Review

The risk of bias according to the domains recommended by the Cochrane Collaboration was evaluated with the following results (Fig. 3). Although some studies were at risk of bias because they had not been blinded and although in the publications the reasons for this were not found, it was considered satisfactory to keep them for the analysis considering that they all fulfilled the condition of adequate random assignment.

Five studies reported results at 48 and/or 96 weeks and two studies had reported results only at 48 weeks at the time of the review. In all studies the random assignment was made on the basis of nucleoside analog reverse-transcriptase inhibitors. Three studies were open-label trials in terms of blinding; others reported double-blind allocation and follow-up. All reported intention-to-treat analysis in their methods and reported the causes of missing data. Three studies assigned efavirenz (EFV) as the third drug in both branches. One study randomly assigned TDF/FTC or ABC/3TC to EFV or atazanavir/ritonavir (ATV/r). A further study assigned ATV/r as the third drug. Finally, one study assigned lopinavir /ritonavir (LPV/r) as the third medication and one more randomized assignment with the entire treatment schedule ABC/3TC plus dolutegravir (DTG) or TDF/FTC plus EFV.

Table 1 presents the baseline characteristics of the patients for each study.

**Table 1:** Baseline characteristics of patients in each of the studies included.

Study	Publication	Medications used	Number of patients assigned		Age (Median)	Mean (Percentage)	Viral Load (log10)	CD4 (count/µL)
ABC/3TC vs. TDF/FTC 1	Moyle G, Europe, 2013 ^31^	ABC/3TC or TDF/FTC. All accompanied by EFV	ABC/3TC	195	38 (19-70)	83	5.01 (2.88-6.78)	240 (10-610)
	Post F, Europe, 2010 ^32^							
			TDF/FTC	197	36 (18-66)	80	5.12 (3.31-6.75)	230 (10-600)
	Stellbrink H, Europe, 2010 ^20^							
ABC/3TC vs. TDF/FTC 2	Nishijima T, Japan, 2013 ^33^	ABC/3TC or TDF/FTC. All accompanied by ATV/r	ABC/3TC	54	39 (28.8-44)	98	4.29 (3.92-4.67)	236 (194-301)
			TDF/FTC	55	35 (29-42)	98	4.28 (3.86-4.6)	269 (177-306)
ABC/3TC vs. TDF/FTC 3	Sax P. United States 2009 ^34^	ABC/3TC or TDF/FTC accompanied by EFV or ATV/r	ABC/3TC Efavirenz	465	37 (31-45)	79	4,7 (4.3 - 5.0)	225 (103-324)
	Daar E. United States, 2011 ^35^		ABC/3TC Atazanavir/ritonavir	463	38 (30-45)	84	4.6 (4.3 - 5.1)	236 (72-346)
	Wyatt C, United States, 2014 ^36^		TDF/FTC Efavirenz	464	39 (31-44)	85	4.7 (4.4-4.9)	234 (103-334)
	McComsey G, United States, 2011 ^37^		TDF/FTC Atazanavir/ritonavir	465	39 (31-46)	83	4.7 (4.3-5.1)	224 (87-327)
ABC/3TC vs. TDF/FTC 4	Walmsley S. North America, Europe, Australia. 2013 ^38^	ABC/3TC and Dolutegravir		422	36 (18-68)	84	4.67	334
		TDF/FTC and Efavirenz		422	35 (18-85)	85	4.70	339
ABC/3TC vs. TDF/FTC 5	Smith K, United States, 2009 ^39^	ABC/3TC and Lopinavir/ritonavir		347	38	84	4.90	214
		TDF/FTC and Lopinavir/ritonavir		347	38	80	4.80	193
ABC/3TC vs. ZDV/3TC	DeJesus E, United States, Europe, South and Central America, 2004 ^40^	ABC or ZDV. All also receiving Lamivudine and Efavirenz	ABC/3TC	327	35 (17-74)	80	4.81 (2.29-5.88)	267 (37-1,883)
			ZDV/3TC	327	35 (20-74)	82	4.76 (1.95-5.88)	258 (25-1,198)
ZDV/3TC vs. TDF/FTC	Nicolas A. Europe, United States. 2009 ^41^	ZDV/3TC or TDF/FTC. All accompanied by EFV	ZDV/3TC	258	37	87	5.00	241
	Pozniak A. Europe, United States. 2006 ^42^							
	Arribas J. Europe, United States. 2008 ^43^		TDF/FTC	259	36	86	5.00	233
	Gallant J. Europe, United States. 2006 ^44^							

'Study' corresponds to the evaluated comparison and 'publication' corresponds to the publications that belonged to the same study. The data, as described in the studies, are presented in medians with their interquartile range. In the boxes where this does not appear, it is due to it not being found in the publication.

### Synthesis of results

The results of the direct comparisons are presented in Tables 2 and ^3^. No differences were found in mortality in any of the comparisons. Only one study reported the outcome of clinical progression to AIDS, without finding differences between ABC/3TC and ZDV/3TC.

The outcomes of treatment adherence and lactic acidosis were not reported in the studies and therefore were not analyzed. Immunological failure was also not found in the studies, although some reported changes in CD4+ T lymphocytes count from baseline values in medians and in other studies, the type of measurement used was not explained, so it was not possible to do an analysis of difference between means.

**Table 2:** Results of Direct Comparisons - Efficacy Outcomes (RR - CI 95%)

	Outcome Studied			
Comparison	Clinical progression to AIDS	Proportion of patients with viral load of <50 copies/mL at 48 weeks	Proportion of patients with viral load of <50 copies/mL at 96 weeks	Virological failure
ABC/3TC vs. TDF/FTC	Data were not found	0.98 (0.91-1.06) I² 78%	0.95 (0.92 - 0.98) I² 1%	1.04 (0.68 - 1.61) I² 79%
ABC/3TC vs. ZDV/3TC	1.6 (0.53 - 4.84)	1.01 (0.91 - 1.12)	Only reported at 48 weeks	1.54 (0.78 - 3.04)
ZDV/3TC vs. TDF/FTC	Data were not found	0.88 (0.79 - 0.98)	0.91 (0.8 - 1.04)	1.18 (0.94 - 1.49)

The I² statistic was calculated only for the ABC/3TC vs. TDF/FTC comparison, whose results are deduced from the meta-analysis using the random effects method. The other comparisons had only one study in the direct comparison and the result is deduced from the data extracted from it.

**Tabla 3:** Results of Direct Comparisons - Safety Outcomes (RR - CI 95%).

	Outcome Studied			
Comparison	Mortality at 96 weeks	Hypersensitivity reactions	Proportion of patients with new cases of acute myocardial infarction or acute cerebrovascular event	Bone marrow suppression
ABC/3TC vs. TDF/FTC	1 (0.99-1.00) I² 20%	1.62 (0.32-8.14) I² 85%	1 (1-1) I² 0%	1 (1 - 1) I² 0%
ABC/3TC vs. ZDV/3TC	No deaths reported	9.33 (2.87-30.4)	1 (0.02-50.25)	0.41 (0.17-0.98)
ZDV/3TC vs. TDF/FTC	1.33 (0.30-5.88)	1.20 (0.53-2.72)	1 (0.02-50.02)	2.36 (1.42-3.92)

The I² statistic was calculated only for the ABC/3TC vs. TDF/FTC comparison, whose results are deduced from the meta-analysis using the random effects method. The other comparisons had only one study in the direct comparison and the result is deduced from the data extracted it.

With respect to the outcomes, lipodystrophy, renal abnormalities and osteopenia/osteoporosis, were not reported in all studies, or it was done with outcome measurement by different laboratory or clinical methods, and in most, the total of patients allocated was not taken into account but was rather just a subpopulation, which we decided not to analyze due to risk of bias by losing the adequate allocation of confounding variables.

### Subgroup analysis

Tables 4, ^5^, ^6^, and ^7^ present the results of the analyzes performed by subgroups, according to the value of basal viral load and the third drug. No meta-analysis were performed because in each comparison there was only one study. In the direct comparisons, no statistically significant differences were found between treatments after differentiation by these subgroups.

**Table 4:** Proportion of patients with viral loads greater than 100,000 copies/mL, the third drug being EFAVIRENZ, presenting the outcome viral load of <50 copies/ml (RR - CI 95%).

Comparison	48 weeks	96 weeks
ABC/3TC vs. TDF/FTC	0.80 (0.64-1.00)	0.90 (0.70-1.15)
ABC/3TC vs. ZDV/3TC	1.00 (0.83-1.19)	Results at 48 weeks
ZDV/3TC vs. TDF/FTC	*	*

* Do not report differentiated events due to viral load

**Table 5:** Proportion of patients with viral loads less than 100,000 copies/mL, the third drug being EFAVIRENZ, presenting the outcome viral load of <50 copies /mL (RR - CI 95%)

Comparison	48 weeks	96 weeks
ABC/3TC vs. TDF/FTC	0.85 (0.70-1.04)	0.83 (0.63-1.09)
ABC/3TC vs. ZDV/3TC	1.01 (0.89-1.15)	Results at 48 weeks
ZDV/3TC vs. TDF/FTC	*	*

* Do not report differentiated events due to viral load

**Table 6:** Proportion of patients with viral loads of less than 100,000 copies/mL, the third drug being ATAZANAVIR/RITONAVIR, presenting the outcome viral load of <50 copies/mL (RR - CI 95%).

Comparison	48 weeks	96 weeks
ABC/3TC vs. TDF/FTC	1.03 (0.86-1.24)	0.94 (0.76-1.16)
ABC/3TC vs. ZDV/3TC	*	*
ZDV/3TC vs. TDF/FTC	*	*

* No data were found to measure the outcome

**Table 7:** Proportion of patients with the third drug LOPINAVIR/RITONAVIR presenting a viral load of <50 copies/ml differentiated by the baseline viral load (RR - CI 95%).

	Viral >100,000 copies/mL		Viral load <100,000 copies/mL	
Comparison	48 weeks	96 weeks	Comparison	48 weeks
ABC/3TC vs. TDF/FTC	0.97 (0.83-1.12)	0.97 (0.81-1.15)	1.03 (0.88-1.19)	1.08 (0.90-1.30)
ABC/3TC vs. ZDV/3TC	*	*	*	*
ZDV/3TC vs. TDF/FTC	*	*	*	*

*There were no comparisons with Lopinavir/Ritonavir

### Indirect comparisons

**Figure 4:**
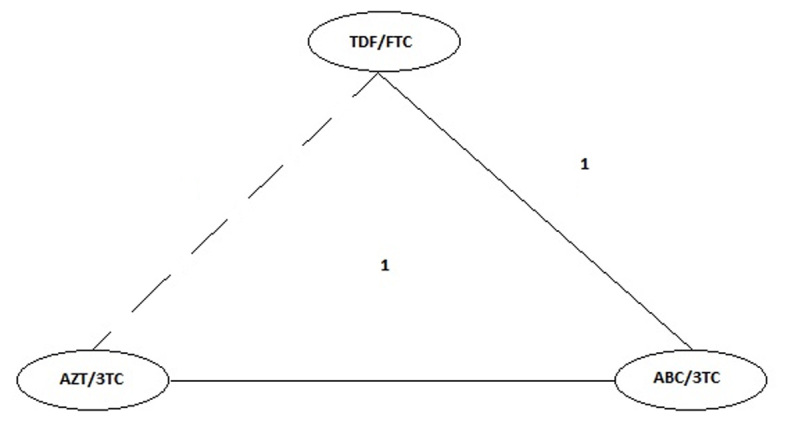
Final Network of Evidence for Meta-analysis. The numbers between the nodes are equivalent to the number of studies that directly compared the interventions joined by the line. The continuous lines correspond to direct comparisons, the dashed line corresponds to the indirect comparison undertaken.

Results could be obtained for an indirect comparison between ZDV/3TC vs. TDF/FTC through the common comparator ABC/3TC when the third drug was EFV, for the outcome ratio of patients with viral load of <50 copies/mL at 48 weeks . For this comparison there were no direct comparisons in this subgroup, so the geometry of the network varies with respect to the one initially proposed (Fig. 4). This comparison met the principle of transitivity in terms of factors modifying the effect of baseline viral load and third drug. As we were unable to perform mixed comparisons in the absence of direct comparisons after subgroup analysis between ZDV/3TC and TDF/FTC, we did not calculate the inconsistency factor. Although the meta-analysis of the direct comparison ABC/3TC vs. TDF/FTC showed high statistical heterogeneity (I² = 78%), in the subgroup analysis, only data from a study with this direct comparison were obtained, which allowed indirect comparisons based on a study which compared ABC/3TC with TDF/FTC and a study comparing ABC/3TC vs. ZDV/3TC (Table 8).

**Table 8:** Indirect comparison between ZDV/3TC and TDF/FTC by means of the common comparator ABC/3TC/EFV for the proportion of patients with the third drug EFAVIRENZ, presenting the outcome viral load of <50 copies/mL at 48 weeks, differentiated by the baseline viral load (RR - CI_95%_).

	Viral load >100,000 copies/mL	Viral load <100,000 copies/mL
Direct comparisons		
ABC/3TC vs. TDF/FTC	0.80 (0.64-1.00)	0.85 (0.70-1.04)
ABC/3TC vs. ZDV/3TC	1.00 (0.83-1.19)	1.01 (0.89-1.15)
Indirect comparison		
TDF/FTC vs. ZDV/3TC	1.25 (0.94-1.67)	1.19 (0.94-1.50)

### Evaluation of reporting biases

In order to control the reporting biases, the search for information was developed according to the established protocol. In addition, a visual inspection of bias in the report was performed by means of a funnel plot for each outcome (figures are attached in Appendix 2), observing symmetry in all of them but not being able to perform tests for asymmetry for the number of included studies, between 4 to 5 in each meta-analysis performed on the direct comparison ABC/3TC vs. TDF/FTC.

## Discussion

### Summary of Evidence

The results obtained in this meta-analysis suggest significant differences in effectiveness outcomes (proportion of patients with viral load of <50 copies/mL) when direct comparisons were evaluated between ABC/3TC vs. TDF/FTC at 96 weeks or ZDV/3TC vs. TDF/FTC at 48 weeks, in favor of TDF/FTC. However, all comparisons were assessed by subgroup analysis, as the review of baseline characteristics in each study made it clear that there were differences in the third drug used, which generated clinical heterogeneity that did not allow for these results to be initially concluded as valid.

Similarly, reports of studies undertaken with the evaluated drugs stated that there were differences in outcomes according to the level of viral load of the patient, prior to the start of treatment.

These analyzes showed that in the direct comparisons of ABC/3TC vs. TDF/FTC and ABC/3TC vs. ZDV/3TC, when the third drug was EFV (with viral load higher and lower than 100,000 copies/mL), there were no significant differences in the outcome of virological response at 48 and 96 weeks. These comparisons allowed an indirect comparison between TDF/FTC and ZDV/3TC at 48 weeks, which also did not report significant differences in the same outcome at 48 weeks.

Likewise, no significant differences were found between ABC/3TC and TDF/FTC when the third drug was ATV/r or LPV/r. Only one study reported a group exposed to DTG as the third drug without being able to obtain a comparator.

The way that outcomes were presented in one of the studies (ABC/3TC vs. TDF/FTC 3) did not allow for the number of patients exposed and with HIV to be extracted, which could have increased the total of patients and hence obtained other conclusions or given greater support to those already obtained.

Significant differences were observed in terms of some adverse effects, specifically bone marrow suppression which affects ZDV/3TC by having a higher risk of presenting this effect in comparison to TDF/FTC or ABC/3TC or hypersensitivity reactions with ABC/3TC versus ZDV/3TC.

Network meta-analysis are supported for their proper development in the random assignment of clinical trials comparing medical interventions directly. This compromises an assumption of similarity, that studies should only be combined if they are considered to be clinically and methodologically similar. In network meta-analysis, covariates that act as effect modifiers should be similar across studies, direct evidence should be consistent with indirect evidence and the evaluation of this assumption should be based initially on clinical judgment on how the differences affect the validity of these types of comparisons ^30^. Unfortunately, with the data available and the lack of compliance with the assumptions of transitivity and homogeneity, it was only possible to make an indirect comparison for just one of the outcomes studied.

We analyzed the possibility of evaluating heterogeneity between the studies using meta-regression and sensitivity analysis, however, taking into account the scarcity of data when decreasing the number of potentially comparable studies according to the effect modifying factors and the variability in the measures used in each study, it was not necessary to explore it beyond the analysis of subgroups as only one or no study remained for each comparison.

### Limitations

Although there are a large number of studies evaluating the interventions analyzed in this review, few met the eligibility criteria to be included in this meta-analysis of controlled clinical trials, and they all had heterogeneity in the selection of the third drug and in the outcomes studied or the definition thereof. Likewise, potential biases were found, specifically regarding the blinding of evaluators or investigators of patient allocation.

It is important to keep in mind that the studies included in the review have results at 48 and 96 weeks, which does not give information beyond this time. Having this information is fundamental for a disease in which the person who suffers it intends to maintain control of it and have an adequate quality of life for many years.

At the same time, the studies reported their outcomes with types of measures that varied between them or with definitions that were not similar in some outcomes, and this limited the extraction of data for statistical analyzes. Furthermore, a broad number of outcomes were proposed to evaluate the safety of the reviewed treatment schedules, which generated several negative outcomes as insufficient information was found in the included studies. Those that did corresponded to sub-studies with a number of patients less than those who received the random assignment in the initial study, and without being able to take these results in a valid form, adequate distribution of confounding variables between the groups could not be assured. From this limitation, new meta-analysis of individual data, with the request and authorization of the authors of the studies, was proposed, in order to standardize the outcome measures and to obtain data with less heterogeneity and to be able to explore the same factor using techniques such as meta-regression and/or sensitivity analysis.

Another limitation is that the current treatment guidelines establish, as a recommended third medication, drugs different from those presented in this review and although this recommendation is based on randomized clinical trials, this allocation was performed with the third drug, leaving the allocation of the inhibitors of reverse-transcriptase at the discretion of the evaluator, which meant they could not be included in this review in an attempt to avoid potential allocation bias and confounding variables.

Finally, all the studies had as baseline the carrying out of genotyping prior to the start of treatment, an examination provides information on if drug resistance mutations are present and based on this; provide the patient with a treatment schedule that has a low probability of failure by virus resistance. In Colombia, the current guidelines do not contemplate conducting this examination at this time, but rather when the first virological failure occurs, a situation that limits the generalizability of the results of the studies analyzed in our country or in countries where the same situation occurs.

## Conclusions

We consider that although the results obtained do not show differences between the treatment schedules evaluated in terms of efficacy outcomes, the limitations discussed previously do not allow these data to be definitive. The results raise the need for further studies, such as clinical trials, to help improve treatment recommendations in HIV-infected patients, with an adequate distribution of the effect modifying factors among the groups compared, as well as evaluating a new systematic review with meta-analysis at the level of the individual data, with the respective permission from the researchers of the included studies or model the results for the future time by means of suitable statistical techniques.

## Figures and Tables

**Figure:**
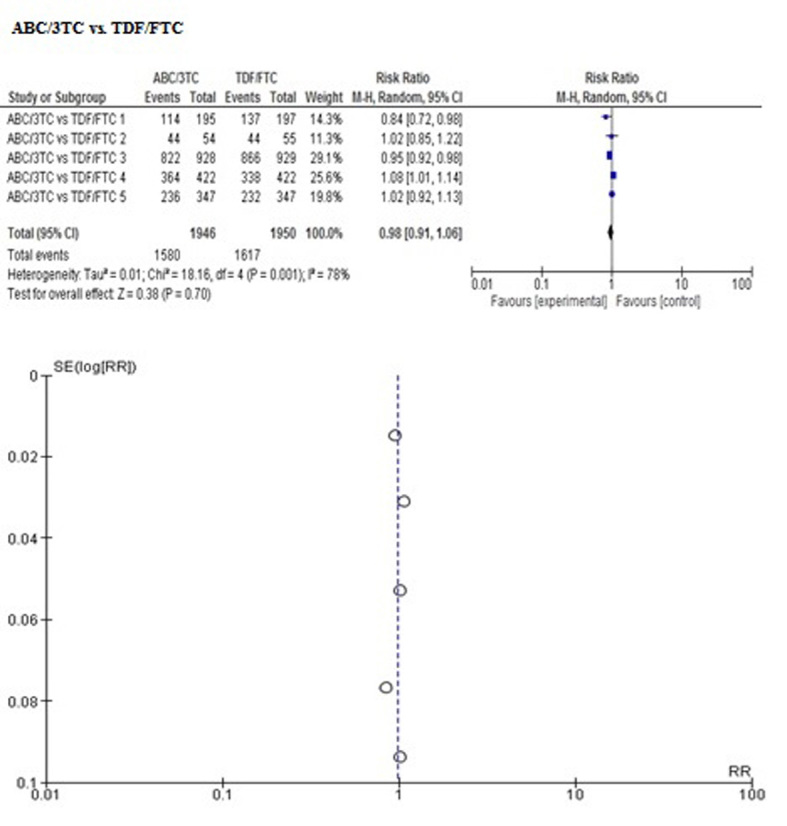
5. Proportion of patients with a viral load of <50 copies/ml at 48 weeks.

**Figure 6:**
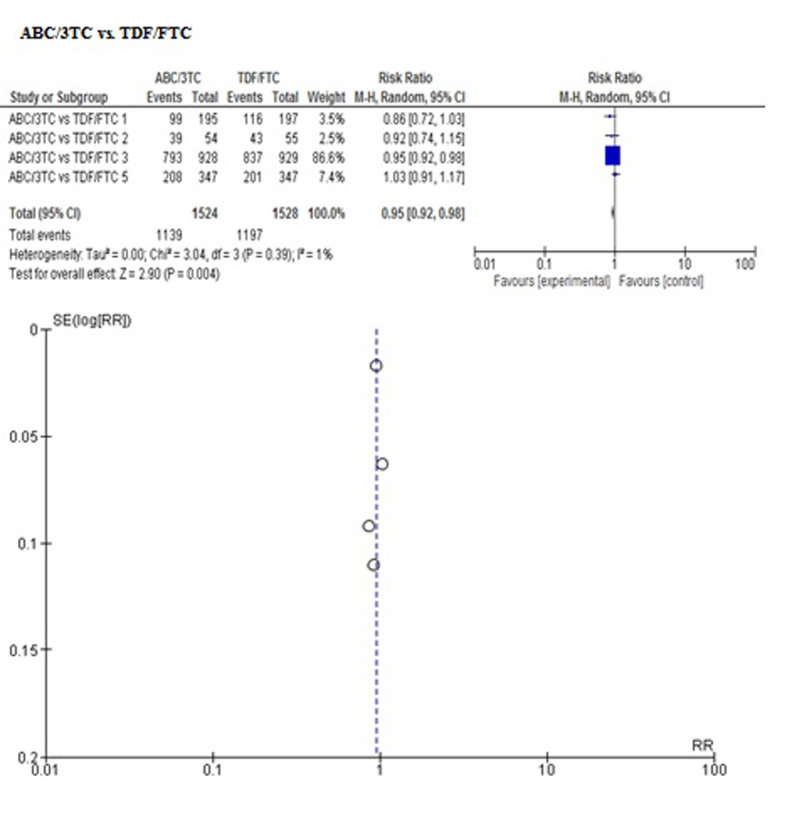
Proportion of patients with a viral load of <50 copies/ml at 96 weeks.

**Figure 7:**
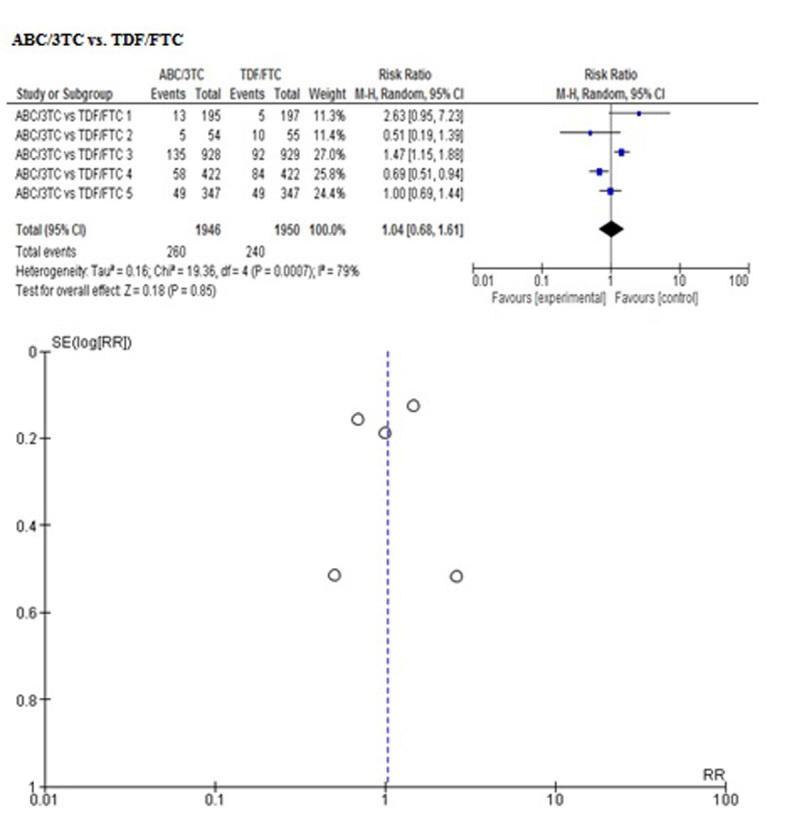
Virological Failure

**Figure 8:**
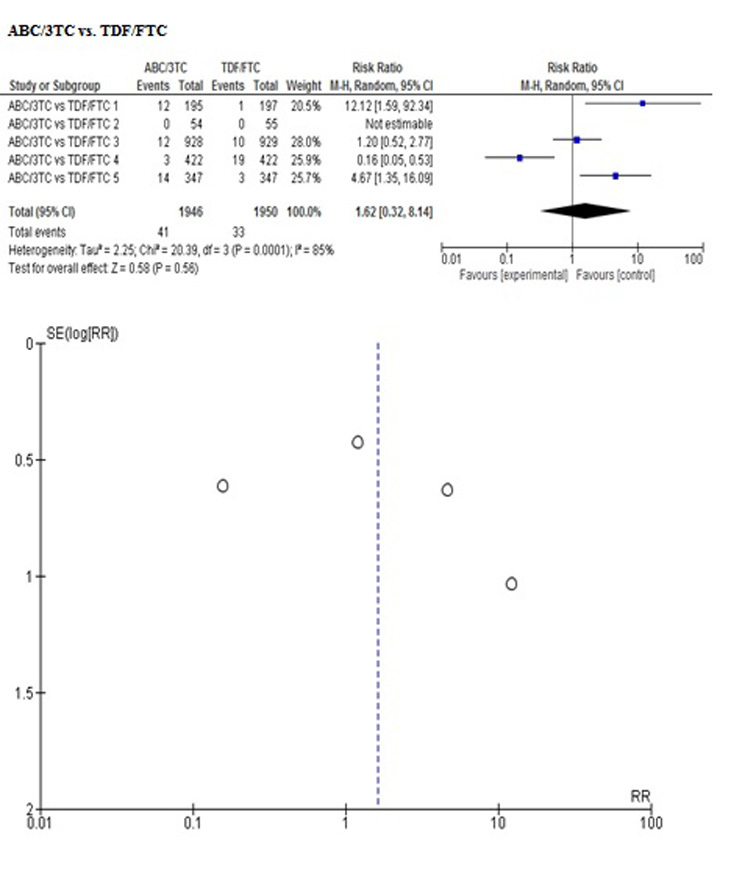
Hypersensitivity Reactions

**Figure 9:**
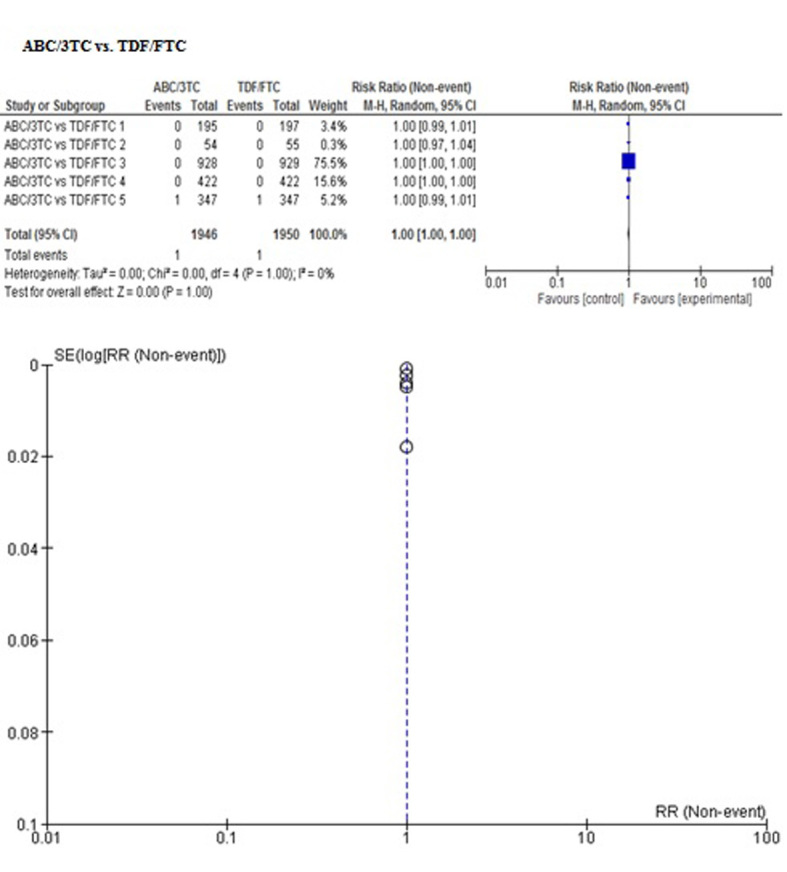
Bone Marrow Suppression

## Appendix 1

**Table 9:** Search strategy

PubMed Search Line	
1	HIV(MeSH) OR HIV-1(MeSH) OR HIV Infections(MeSH)
2	Abacavir(tiab) OR lamivudine(tiab) OR tenofovir(tiab) OR emtricitabine(tiab) OR zidovudine(tiab)
3	1 and 2
4	3 and systematic(sb)
5	HIV-1(MeSH)
6	5 and 2
7	6 AND (randomized controlled trial(pt) OR controlled clinical trial(pt) OR randomized(tiab) OR placebo(tiab) OR drug therapy(sh) OR randomly(tiab) OR trial(tiab) OR groups(tiab)) NOT (animals (mh) NOT humans (mh))
EMBASE Search Line	
	'human immunodeficiency virus'/exp and ('abacavir'/exp or 'lamivudine'/exp or 'tenofovir'/exp or 'emtricitabine'/exp or 'zidovudine'/exp) and ((cochrane review)/lim or (systematic review)/lim or (controlled clinical trial)/lim or (randomized controlled trial)/lim or (metaanalysis)/lim)
Cochrane Search Line	
	HIV + (abacavir OR lamivudine OR tenofovir OR emtricitabine OR zidovudine)
