# Supplementary material for: Zidovudine/Lamivudine vs. Abacavir/Lamivudine vs. Tenofovir/Emtricitabine in fixed-dose combinations as initial treatment for HIV patients: a systematic review and network meta-analysis
Source: Colomb Med (Cali). 2017 Jun 30;48(2):70–81. (PMC5625561)
Supplement: Translation

# Translation: Zidovudina/Lamivudina vs Abacavir/Lamivudina vs Tenofovir/Emtricitabina en combinaciones de dosis fija como tratamiento inicial en pacientes con VIH: revisión sistemática y metanálisis en red

## Introducción

Han pasado más de 30 años desde el reporte de cinco jóvenes homosexuales con una rara enfermedad, neumonía por *Pneumocystis carinii*, en tres centros hospitalarios de Los Angeles, Estados Unidos ^1^. Varios eventos ocurrieron desde entonces hasta la definición del Síndrome de Inmunodeficiencia Adquirida (SIDA) en 1982 ^2^. No pasó mucho tiempo para que los pocos casos iniciales se convirtieran en decenas de millones, generando una de las más grandes pandemias de los tiempos modernos ^3^.

Para el tratamiento de la infección por VIH se han aprobado 26 medicamentos los cuales han disminuido la mortalidad por la infección. La reducción en los costos iniciales de los medicamentos antirretrovirales, la disponibilidad de medicamentos genéricos y el incremento del financiamiento internacional han marcado la expansión de su uso en países de bajos y medianos ingresos ^3^.

Diferentes sociedades en diversos contextos de sistemas de salud, han emitido guías de tratamiento antirretroviral en adultos y adolescentes, con actualización periódica de sus recomendaciones. En la Guía de la Sociedad Europea de SIDA, la de la Organización Mundial de la Salud (OMS) y la de la Sociedad Antiviral Internacional (Estados Unidos), se definen recomendaciones para los esquemas de inicio preferidos, con algunas diferencias entre ellas, pues la guía de la OMS aclara que en los casos en que no se pueda utilizar Tenofovir Disoproxil Fumarato/Emtricitabina (TDF/FTC), se puede utilizar Zidovudina/Lamivudina (AZT/3TC) y que los regímenes que incluyan Abacavir (ABC) no se consideran alternativas preferidas. La Guía Europea establece como esquemas preferidos aquellos que contengan TDF/FTC o Abacavir/Lamivudina (ABC/3TC), aclarando que este último debe utilizarse con precaución en casos con cargas virales mayores de 100,000 copias/mL); finalmente el panel estadounidense recomienda varios esquemas como regímenes iniciales, cada uno con una indicación o una advertencia sobre su uso dependiendo de la carga viral basal, el tercer fármaco escogido en el régimen y las condiciones específicas del paciente que contraindiquen el uso de uno o varios de los medicamentos disponibles, aclarando que la mayoría de los esquemas contienen TDF/FTC o ABC/3TC como columna vertebral del tratamiento ^4^^-^^6^. 

En Colombia la atención de los pacientes se hizo con base en la Guía para el manejo del VIH/SIDA basada en la evidencia. En esta se recomendaban la Zidovudina (AZT) más Lamivudina (3TC) como los inhibidores de transcriptasa reversa análogos de nucleósido preferidos ^7^^,^^8^. En la actualización de 2014 de esta Guía, el régimen AZT/3TC pasó a ser recomendado como régimen alternativo ^9^. En otros países de Latinoamérica, como Argentina y Chile las tres combinaciones se mantienen dentro de los regímenes recomendados para el inicio de la terapia ^10^^,^^11^.

Lo anterior deja claro que existen en el mundo dos regímenes de inhibidores de transcriptasa reversa análogos de nucleósidos preferidos para incluir en los esquemas de inicio de tratamiento antirretroviral (TAR) y que en algunos países, la opción de AZT/3TC sigue siendo contemplada como recomendada o alternativa

Los resultados de las comparaciones directas de la eficacia y seguridad de los regímenes mencionados han mostrado una efectividad similar entre regímenes que contienen TDF/FTC vs AZT/3TC, con diferencias en cuanto a la seguridad de los regímenes, incluso diferenciados por género ^12^^,^^13^. Otros estudios han encontrado que medicamentos como 3TC y FTC son clínicamente equivalentes ^14^. La comparación de las combinaciones de ABC/3TC vs TDF/FTC en algunos estudios mostró una eficacia antiviral similar ^15^^,^^16^, mientras que en otro se encontró mayor riesgo de falla virológica en los que utilizaron ABC/3TC cuando la carga viral es mayor de 100,000 copias/mL ^17^.

En cuanto a la seguridad de los diferentes esquemas, también existen resultados divergentes. Se han reportado diferencias en cuanto a efectos secundarios renales, con incremento en marcadores de disfunción tubular en pacientes que recibieron TDF/FTC ^18^^,^^19^, sin dejar clara la relevancia clínica de tales hallazgos. De igual manera se observó un mayor impacto en la densidad ósea con los esquemas basados en TDF/FTC comparados con los esquemas basados en ABC/3TC ^20^^,^^21^.

No existen comparaciones que incluyan estos tres regímenes, por lo que el objetivo de esta investigación fue comparar la eficacia y seguridad de la combinación de AZT/3TC vs ABC/3TC vs TDF/FTC como integrantes del tratamiento antirretroviral de gran actividad (TARGA) en pacientes mayores de 18 años con infección por VIH e indicación de inicio de tratamiento.

Las revisiones sistemáticas de ensayos clínicos con asignación aleatoria se consideran el estándar en las decisiones del cuidado de la salud basadas en la evidencia. Muchas revisiones sistemáticas utilizan metanálisis para combinar resultados cuantitativos y resumir la evidencia disponible. De esta manera, un metanálisis puede mejorar el conocimiento acerca de una estrategia terapéutica aumentando la potencia estadística, la precisión en el tamaño del efecto del tratamiento o resolviendo controversias que surgieron de estudios aparentemente contradictorios. Sin embargo, pueden tener limitaciones que pueden afectar la validez de los resultados obtenidos, en el caso de combinar estudios con características clínicas diferentes entre los participantes en los mismos, o con resultados erróneos si se combinan estudios con riesgo de sesgo, generando un resumen inapropiado. Estas limitaciones deben tenerse en cuenta previo a la realización del metanálisis para un adecuado manejo de dichas limitaciones que lleven a obtener resultados válidos y generalizables ^22^. Idealmente los ensayos clínicos deberían comparar simultáneamente todas las intervenciones de interés; sin embargo, tales estudios casi nunca están disponibles. En ausencia de estudios que comprendan una comparación directa, una comparación indirecta podría proveer evidencia útil. De igual manera, la combinación de evidencia directa e indirecta puede fortalecer la evaluación de las intervenciones disponibles ^23^.

## Materiales y Métodos

Se realizó una revisión sistemática y metanálisis en red en la que se incluyeron ensayos clínicos paralelos con asignación aleatoria, realizados con cualquier propósito (equivalencia, superioridad, no inferioridad), en un solo centro o multicéntricos, en cualquier idioma, en cualquier país y con cualquier tiempo de seguimiento. Se incluyeron estudios realizados con pacientes mayores de 13 años, con diagnóstico confirmado de infección por VIH por medio de cualquier prueba confirmatoria directa o indirecta, en quienes se decidió iniciar TARGA y que no estuvieran expuestos previamente en forma directa a ningún medicamento antirretroviral. 

Las intervenciones que se compararon fueron las coformulaciones de TDF/FTC (300/200 mg cada 24 h vía oral), ABC/3TC (600/300 mg cada 24 h vía oral) y AZT/3TC (300/150 mg cada 12 h vía oral) que se hubieran realizado en comparación directa entre ellas o con placebo.

### Medidas de desenlace 

Los desenlaces fueron: i) mortalidad, ii) progresión clínica a SIDA (proporción de pacientes que en los estudios presentan una enfermedad definitoria de SIDA o progresan al estadio C y/o 3 de la clasificación de los Centros para el control de enfermedades de Atlanta, USA (CDC) de 1993 o 2008, luego de iniciar el tratamiento antirretroviral) ^24^^,^^25^, iii) respuesta virológica al tratamiento antirretroviral definida como la proporción de pacientes que alcanzaron una carga viral menor a 50 copias/mL a las 48 y 96 semanas, luego de iniciar el tratamiento antirretroviral ^6^, iv) falla virológica (carga viral de VIH > 50 copias/mL 6 meses después de iniciar la terapia en personas que se mantienen en tratamiento antirretroviral) ^6^, v) adherencia al tratamiento (Proporción de pacientes que al finalizar el tiempo del estudio continúan con el mismo esquema inicial sin interrupciones del mismo), vi) falla inmunológica (conteo de CD4 que cae desde la línea de base o es inferior a 100 células/µL persistentemente) ^5^, vii) reacción de hipersensibilidad a ABC (síndrome clínico multiorgánico que se presenta en las primeras 6 semanas luego de iniciar tratamiento con ABC) ^4^, viii) proporción de nuevos casos de Infarto Agudo de miocardio (IAM) o Evento cerebrovascular agudo (ECV) luego del inicio del tratamiento antirretroviral, ix) supresión de médula ósea definida como la proporción de nuevos casos de anemia y/o neutropenia luego del inicio del tratamiento antirretroviral, x) acidosis láctica (aumento del lactato sérico >5 mmol/L asociado a síntomas sistémicos) ^6^, xi) lipodistrofia (Aumento o disminución de la grasa subcutánea medida por antropometría o absorciometría de rayos x de energía dual - DXA) (26), xii) anormalidades renales (tubulopatías, nefrolitiasis, nefritis intersticial) ^6^, y xiii) osteopenia (mujer posmenopaúsica u hombre con ≥ 50 años con densidad mineral ósea medida por T-score en DXA -1 a -2.5) osteoporosis (mujer posmenopaúsica u hombre con ≥50 años con densidad mineral ósea medida por T-score en DXA ≤-2.5 o mujer premenopaúsica u hombre con <50 años con densidad mineral ósea medida por Z-score en DXA ≤-2 y fracturas por fragilidad) ^6^.

### Métodos de búsqueda para la identificación de estudios 

Todas las búsquedas se realizaron sin restricción de idioma o país. Se limitaron a estudios en humanos, desde 1995 (inicio de la TARGA) hasta mayo de 2014.

Se hizo una búsqueda electrónica en las siguientes bases de datos: MEDLINE, EMBASE, Registro Central Cochrane de Ensayos Controlados (CENTRAL), Base de datos Cochrane de Revisiones Sistemáticas, Literatura Latinoamericana de Ciencias de la Salud (LILACS), Índice Médico Africano, Plataforma de registros internacionales de Ensayos Clínicos de la Organización Mundial de la Salud (OMS), Clinical Trials. Además se buscaron los resúmenes, pósteres, conferencias presentadas en los congresos sobre retrovirus e infecciones oportunistas (CROI), conferencias internacionales del VIH, AIDS Clinical Trials Group Network (ACTG). Se hizo búsqueda en tablas de contenido de las siguientes revistas: New England Journal of Medicine, Journal of American Medical Association (JAMA), LANCET, Journal of the International AIDS Society, Official Journal of the International AIDS Society. Se realizó revisión de las referencias de todos los estudios encontrados.

Los términos utilizados en la búsqueda fueron "abacavir", "lamivudine", "tenofovir", "emtricitabine", "zidovudine", "randomized controlled trial", "controlled clinical trial", "randomized", "placebo", "drug therapy", "randomly", "trial", "groups" (Anexo 1).

### Selección de estudios 

La selección final de los estudios la hicieron dos revisores de manera independiente, ambos expertos en la atención de pacientes con VIH en Colombia y estudiantes de Epidemiología, con la asesoría de una bibliotecóloga experta en búsqueda en bases de datos de las ciencias de la salud.

Ambos revisores evaluaron todos los títulos y resúmenes y excluyeron aquellos que se consideraron irrelevantes para la revisión, por no cumplir los criterios de inclusión o por duplicación. Posteriormente realizaron evaluación del texto completo de los estudios para verificar los criterios de elegibilidad. El acuerdo entre los dos revisores se evaluó mediante estadístico kappa simple, resolviendo los desacuerdos mediante discusión entre ambos.

### Extracción y manejo de las variables 

Se extrajeron todas las variables que se consideraron relevantes para la comparación de los estudios y medición de los desenlaces, pertenecientes a los siguientes tipos: fuente, elegibilidad, métodos, participantes, intervenciones, desenlaces, resultados, fuente de financiación.

### Evaluación del riesgo de sesgo en los estudios incluidos 

Se realizó mediante los criterios recomendados por la Colaboración Cochrane ^22^ y mediante el programa Review Manager 5.3, que incluyen revisión de la generación de la secuencia de asignación aleatoria, ocultamiento de la asignación, cegamiento del personal participante y los pacientes, cegamiento de la evaluación del desenlace, reporte y manejo de los datos perdidos, reporte selectivo, otros sesgos potenciales. 

### Geometría de la red

Se definió de acuerdo con las comparaciones directas encontradas en los estudios elegidos luego de cumplir con los criterios de elegibilidad, revisión por evaluadores. Si entre todos los estudios incluidos existió al menos una comparación directa entre cada uno de los tratamientos evaluados, se definió que la geometría de la red correspondió a un asa cerrada ^23^. 

### Medidas del efecto del tratamiento 

Luego de analizar el tipo de desenlaces estudiados, que corresponden a proporciones (número de pacientes con el evento comparado frente al número total de pacientes asignados a cada tratamiento) y de analizar las ventajas y desventajas de cada medida del efecto, y teniendo en cuenta que las medidas relativas son más consistentes que las medidas absolutas, así como la facilidad de interpretación por los clínicos ^22^, se definió que la medida más consistente con nuestros desenlaces es el riesgo relativo, con su respectivo intervalo de confianza del 95%. Al realizar la extracción de los datos, cuando se encontró un desenlace reportado con medidas continuas, se analizó si era posible extraer las medias y las desviaciones estándar con el fin de analizar mediante diferencia de medias. 

### Métodos de análisis

#### Síntesis de los datos 

La estimación del efecto de cada desenlace se realizó inicialmente mediante metanálisis de comparaciones directas, con el fin de obtener el efecto de cada combinación y poder utilizarlo posteriormente en el metanálisis en red. Para tal efecto, teniendo en cuenta que los estudios podían haber reportado una cantidad escasa de sucesos y que el método genérico de inverso de la varianza puede ser menos robusto en este contexto, se utilizó el método de Mantel - Hanzel para resultados dicotómicos, con riesgo relativo como medida del efecto. De igual manera se analizaron las comparaciones mediante análisis de efectos aleatorios, pues debido a condiciones como la variabilidad del tercer medicamento entre estudios, no se puede concluir que no haya variación entre los tamaños del efecto entre los estudios. En cada medición se realizó evaluación de la heterogeneidad clínica, mediante investigación y comparación de la diversidad en las características basales de los participantes (intervenciones comparadas, edad, género, carga viral, recuento de linfocitos T CD4^+)^ incluidos en cada estudio y de la heterogeneidad estadística (variabilidad en los efectos de la intervención) mediante el estadístico I². Cuando se encontró que existía heterogeneidad clínica o estadística (I² mayor del 40%) se evaluó mediante análisis de subgrupos por las variables consideradas modificadoras del efecto, definidas como aquellas características de los pacientes o de los estudios que pueden estar asociadas con el efecto final del tratamiento ^27^ y si dichas variables eran homogéneas entre las comparaciones evaluados, se definió realizar un análisis estadístico mediante metanálisis ^22^. 

El total de pacientes expuestos en cada desenlace correspondió al total de pacientes asignados aleatoriamente a cada comparación, puesto que los análisis en cada estudio fueron por intención a tratar y con el fin de conservar la asignación aleatoria como supuesto para realizar las comparaciones directas e indirectas

#### Comparaciones indirectas

De acuerdo con la geometría de la red encontrada y teniendo a priori la posibilidad que fuera de asa cerrada, con mínimo una comparación directa disponible entre dos intervenciones, se propuso realizar el análisis final para cada desenlace mediante el método de comparaciones mixtas o el método de comparaciones indirectas de Bucher, los cuales tienen como supuesto fundamental que las comparaciones ocurren a través de un asa cerrada para las comparaciones mixtas y que la eficacia relativa de un tratamiento es la misma en todos los estudios incluidos en la comparación indirecta para el método de Bucher. Además, para que sus resultados sean válidos, el efecto de cualquier tratamiento debe ser intercambiable a través de los demás estudios de la red. Este método tiene la fortaleza de preservar la asignación aleatoria ^28^.

Existen diversos programas estadísticos que permiten realizar este tipo de comparaciones y para el caso de comparaciones con redes como la nuestra con 3 intervenciones, donde los estudios individuales son comparaciones de dos brazos, se han diseñado métodos sencillos en hojas de Excel para la obtención de los resultados ^29^

#### Evaluación de la inconsistencia

Para realizar las comparaciones indirectas es fundamental que se cumplan los principios de transitividad, que son la similitud en cuanto a las variables modificadoras del efecto y la consistencia entre los estudios, definida como el acuerdo entre las fuentes directas e indirectas de la evidencia y que se evalúa estadísticamente mediante el factor de inconsistencia a través de la aproximación específica de asa.

#### Evaluación de sesgos de reporte 

Para evaluar la presencia de sesgos se utilizaron las siguientes estrategias: gráfico de embudo y prevención de los sesgos de idioma, publicación duplicada, ubicación, citación, mediante el cumplimiento estricto de la metodología diseñada para la revisión sistemática. 

#### Análisis de subgrupos 

De acuerdo con el conocimiento previo y reportes en estudios, se consideró que las principales causas de heterogeneidad en los efectos de cada tratamiento, definidos igualmente como modificadores del efecto, son el nivel de carga viral y el tercer fármaco. Por tal motivo, se realizaron análisis de subgrupos de acuerdo con el nivel basal de carga viral antes del inicio del tratamiento, evaluando las diferencias en los efectos entre los subgrupos con cargas virales mayores o iguales a 100,000 copias/mL y menores de 100,000 copias/mL y la respuesta diferencial de acuerdo al tercer fármaco (inhibidor de transcriptasa reversa no nucleósido, inhibidor de proteasa o inhibidor de integrasa)

## Resultados

### Selección de estudios

La búsqueda identificó 5,152 títulos para su evaluación inicial, de los que se excluyeron 4,936 luego de la revisión del título, resumen y posible duplicación. Fueron excluidos 2,711 debido a que la terapia evaluada no correspondía a la incluida en la revisión, 1,963 evaluaban desenlaces no estudiados en nuestra revisión, 89 fueron estudios realizados en niños, 74 en gestantes, 40 estudiaban tratamientos para otra condición patológica asociada al VIH, 10 correspondieron a estudios de diagnóstico y se detectaron 49 títulos duplicados entre las bases de datos. Los 216 artículos restantes fueron revisados en su texto completo verificando criterios de elegibilidad. Luego de esta revisión, se obtuvieron 15 publicaciones correspondientes a 7 estudios, 5 comparando ABC/3TC vs TDF/FTC, uno AZT/3TC vs TDF/FTC y uno ABC/3TC vs AZT/3TC (Fig. 1).

**Figura 1:** Flujograma con los resultados de la búsqueda

El acuerdo entre los dos revisores se evaluó mediante un estadístico kappa simple, obteniendo un kappa de 0.9239 (IC 95%: 0.81-1.00). Los dos desacuerdos entre los revisores se resolvieron mediante discusión entre los mismos y no hubo necesidad de acudir a un tercer revisor.

### Geometría de la red

Luego de definir los estudios incluidos en el metanálisis se obtuvo la siguiente red de comparaciones directas, el número indica la cantidad de estudios entre cada nodo (Fig. 2)

**Figura 2:** Red de evidencia para el metanálisis. Los números entre los nodos equivalen a la cantidad de estudios que compararon en forma directa las intervenciones unidas por la línea

### Evaluación del riesgo de sesgo en los estudios incluidos 

Se evaluó el riesgo de sesgos de acuerdo a los dominios recomendados por la colaboración Cochrane, con los siguientes resultados (Fig. 3). A pesar que algunos estudios tenían riesgo de sesgos porque no habían sido cegados y aunque en las publicaciones no se encontraron las razones de ello, se consideró dejarlos para el análisis teniendo en cuenta que cumplieron la condición de asignación aleatoria adecuada en todos ellos. 

**Figura 3:** Riesgo de sesgo en estudios incluidos en el metanálisis.

Cinco estudios reportaron resultados a 48 y/o 96 semanas y dos estudios tenían reporte de resultados sólo a las 48 semanas al momento de realizar la revisión. En todos los estudios la asignación aleatoria se hizo con base en los inhibidores de transcriptasa reversa análogos de nucleósido. Tres estudios fueron de etiqueta abierta en cuanto a su cegamiento, los demás reportaron doble ciego en la asignación y seguimiento. Todos reportaron en sus métodos análisis por intención a tratar y reportaron las causas de los datos perdidos. Tres estudios asignaron como tercer medicamento a Efavirenz (EFV) en ambos brazos. Un estudio asignó aleatoriamente, además de TDF/FTC o ABC/3TC a EFV o Atazanavir/ritonavir (ATV/r). Un estudio más asignó como tercer medicamento a ATV/r. Finalmente, un estudio asignó como tercer medicamento a Lopinavir/ritonavir (LPV/r) y uno más realizó la asignación aleatoria con el esquema completo, ABC/3TC más Dolutegravir (DTG) o TDF/FTC más EFV.

En la Tabla 1 se presentan las características basales de los pacientes para cada estudio

**Tabla 1:** Características basales de los pacientes en cada uno de los estudios incluidos.

Estudio	Publicación	Medicamentos utilizados	Número pacientes asignados		Edad (Mediana)	Hombres (Porcentaje)	Carga viral (Log10)	CD4 (recuento/µL)
ABC/3TC vs TDF/FTC 1	Moyle G, Europa, 2013 (31)	ABC/3TC o TDF/FTC. Todos acompañados con EFV	ABC/3TC	195	38 (19-70)	83%	5.01 (2.88-6.78)	240 (10-610)
	Post F, Europa, 2010 (32)							
			TDF/FTC	197	36 (18-66)	80%	5.12 (3.31-6.75)	230 (10-600)
	Stellbrink H, Europa, 2010 (20)							
ABC/3TC vs TDF/FTC 2	Nishijima T, Japón, 2013 (33)	ABC/3TC o TDF/FTC. Todos acompañados de ATV/r	ABC/3TC	54	39 (28.8-44)	98.1%	4.29 (3.92-4.67)	236.5 (194-301.3)
			TDF/FTC	55	35 (29-42)	98.2%	4.28 (3.86 - 4.6)	269 (177-306)
ABC/3TC vs TDF/FTC 3	Sax P. Estados Unidos, 2009 (34)	ABC/3TC o TDF/FTC acompañados de EFV o ATV/r	ABC/3TC Efavirenz	465	37 (31-45)	79%	4.7 (4.3-5.0)	225 (103-324)
	Daar E. Estados Unidos, 2011 (35)		ABC/3TC Atazanavir/ritonavir	463	38 (30-45)	84%	4.6 (4.3-5.1)	236 (72-346)
	Wyatt C, Estados Unidos, 2014 (36)		TDF/FTC Efavirenz	464	39 (31-44)	85%	4.7 (4.4-4.9)	234 (103-334)
	McComsey G, Estados Unidos, 2011 (37)		TDF/FTC Atazanavir/ritonavir	465	39 (31-46)	83%	4.7 (4.3-5.1)	224 (87-327)
ABC/3TC vs TDF/FTC 4	Walmsley S. Norteamérica, Europa, Australia. 2013 (38)	ABC/3TC más Dolutegravir		422	36 (18-68)	84%	4.67	334.5
		TDF/FTC más Efavirenz		422	35 (18-85)	85%	4.7	339
ABC/3TC vs TDF/FTC 5	Smith K, Estados Unidos, 2009 (39)	ABC/3TC más Lopinavir/ritonavir		347	38	84%	4.9	214
		TDF/FTC más Lopinavir/ritonavir		347	38	80%	4,8	193
ABC/3TC vs AZT/3TC	DeJesus E, Estados Unidos, Europa, Sur y Centro América, 2004 (40)	ABC o AZT. Todos recibiendo además Lamivudina y Efavirenz	ABC/3TC	327	35 (17-74)	80%	4.81 (2.29-5.88)	267 (37-1883)
			AZT/3TC	327	35 (20-74)	82%	4.76 (1.95-5.88)	258 (25-1,198)
AZT/3TC vs TDF/FTC	Nicolas A. Europa, Estados Unidos. 2009 (41)	AZT/3TC o TDF/FTC. Todos acompañados de EFV	AZT/3TC	258	37	87%	5.00	241
	Pozniak A. Europa, Estados Unidos. 2006 (42)							
	Arribas J. Europa, Estados Unidos. 2008 (43)		TDF/FTC	259	rowspan="2"36	86%	5.00	233
	Gallant J. Europa, Estados Unidos. 2006 (44)							

El estudio corresponde a la comparación evaluada y publicación corresponde a las publicaciones que pertenecían al mismo estudio. Los datos, de acuerdo a lo descrito en los estudios se presentan en medianas con su rango intercuartil, en las casillas que este no se presenta se debe a que no se encontró en la publicación

### Síntesis de los resultados

Los resultados de las comparaciones directas se presentan en las Tablas 2 y ^3^. No se encontraron diferencias en cuanto a mortalidad en ninguna de las comparaciones. Sólo un estudio reportó el desenlace de progresión clínica a SIDA, sin encontrar diferencias entre ABC/3TC y AZT/3TC.

**Tabla 2:** Resultados de las comparaciones directas - Desenlaces de eficacia (RR - IC 95%)

	Desenlace estudiado			
Comparación	Progresión clínica a sida	Proporción de pacientes con carga viral <50 copias/mL a las 48 semanas	Proporción de pacientes con carga viral >50 copias/mL a las 96 semanas	Falla virológica
ABC/3TC vs TDF/FTC	No se encontraron datos	0.98 (0.91-1.06) I² 78%	0.95 (0.92-0.98) I² 1%	1.04 (0.68-1.61) I² 79%
ABC/3TC vs AZT/3TC	1.6 (0.53-4.84)	1.01 (0.91-1.12)	Sólo tiene reporte a las 48 semanas	1.54 (0.78-3.04)
AZT/3TC vs TDF/FTC	No se encontraron datos	0.88 (0.79-0.98)	0-91 (0.80 - 1.04)	1.18 (0.94-1.49)

El estadístico I² se calculó sólo para la comparación ABC/3TC vs TDF/FTC, cuyos resultados se deducen de la realización de metanálisis con el método de efectos aleatorios. Las demás comparaciones sólo tenían un estudio en la comparación directa y el resultado se deduce de los datos extraídos del mismo.

**Tabla 3:** Resultados de las comparaciones directas - Desenlaces de seguridad (RR - IC 95%).

	Desenlace estudiado			
Comparación	Mortalidad 96 semanas	Reacciones de hipersensibilidad	Proporción de pacientes con nuevos casos de infarto agudo del miocardio o evento cerebrovascular agudo	Supresión de médula ósea
ABC/3TC vs TDF/FTC	1.00 (0.99-1.00) I² 20%	1.62 (0.32-8,14) I² 85%	1 (1-1) I² 0%	1 (1-1) I² 0%
ABC/3TC vs AZT/3TC	No reportaron muertes	9,33 (2,87 - 30,4)	1.00 (0.02-50.25)	0.41 (0.17-0.98)
AZT/3TC vs TDF/FTC	1,33 (0,3 - 5,88)	1,2 (0,53 - 2,72)	1.00 (0.02-50.02)	2.36 (1.42-3.92)

El estadístico I² se calculó sólo para la comparación ABC/3TC vs TDF/FTC, cuyos resultados se deducen de la realización de metanálisis con el método de efectos aleatorios. Las demás comparaciones sólo tenían un estudio en la comparación directa y el resultado se deduce de los datos extraídos del mismo.

Los desenlaces de adherencia al tratamiento y acidosis láctica no fueron reportados en los estudios por lo cual no se analizaron. La falla inmunológica, tampoco se encontró en los estudios, aunque algunos informaron el cambio en el recuento de linfocitos CD4+ desde el valor basal en medianas y en otros no se explicaba el tipo de medida utilizada, por lo cual no fue posible hacer el análisis de diferencia de medias.

Con respecto a los desenlaces lipodistrofia, anormalidades renales, osteopenia/osteoporosis, tampoco fueron reportados en todos los estudios, o se hizo con medición del desenlace por diferentes métodos de laboratorio o clínicos y en la mayoría no se tuvieron en cuenta al total de los pacientes asignados sino a una subpoblación, que decidimos no analizar por el riesgo de sesgo al perder la asignación adecuada de las variables confusoras. 

### Análisis de subgrupos

En las Tablas 4, ^5^, ^6^y ^7^ se presentan los resultados de los análisis realizados por subgrupos, de acuerdo con el valor de carga viral basal y tercer fármaco. No se realizaron metanálisis porque en cada comparación solo había un estudio. En las comparaciones directas no se encontraron diferencias estadísticamente significativas entre los tratamientos luego de diferenciar por estos subgrupos. 

**Tabla 4:** Proporción de pacientes con carga viral >100,000 copias/mL y tercer fármaco EFAVIRENZ, que presentan el desenlace carga viral <50 copias/mL (RR-IC 95%)

Comparación	48 semanas	96 semanas
ABC/3TC vs TDF/FTC	0.80 (0.64-1.00)	0.90 (0.70-1.15)
ABC/3TC vs AZT/3TC	1.00 (0.83-1.19)	Resultados a 48 semanas
AZT/3TC vs TDF/FTC	*	*

* No reportan eventos diferenciados por carga viral

**Tabla 5:** Proporción de pacientes con carga viral <100,000 copias/mL y tercer fármaco EFAVIRENZ, que presentan el desenlace carga viral <50 copias/mL (RR - IC 95%)

Comparación	48 semanas	96 semanas
ABC/3TC vs TDF/FTC	0.85 (0.70-1.04)	0.83 (0.63-1.09)
ABC/3TC vs AZT/3TC	1.01 (0.89-1.15)	Resultados a 48 semanas
AZT/3TC vs TDF/FTC	*	*

* No reportan eventos diferenciados por carga viral

**Tabla 6:** Proporción de pacientes con carga viral <100,000 copias/mL y tercer fármaco ATAZANAVIR/RITONAVIR, que presentan el desenlace carga viral <50 copias/mL (RR-IC 95%)

Comparación	48 semanas	96 semanas
ABC/3TC vs TDF/FTC	1.03 (0.86-1.24)	0.94 (0.76-1.16)
ABC/3TC vs AZT/3TC	*	*
AZT/3TC vs TDF/FTC	*	*

* No se encontraron datos para medir el desenlace

**Tabla 7:** Proporción de pacientes con tercer fármaco LOPINAVIR/RITONAVIR que presentan el desenlace carga viral <50 copias/mL diferenciados por el nivel de carga viral basal (RR-IC 95%)

	Carga viral >100,000 copias/mL			Carga viral <100,000 copias/mL	
Comparación	48 semanas	96 semanas	Comparación	48 semanas
ABC/3TC vs TDF/FTC	0.97 (0.83-1.12)	0.97 (0.81-1.15)	1.03 (0.88-1.19)	1.08 (0.90-1.30)
ABC/3TC vs AZT/3TC	*	*	*	*
AZT/3TC vs TDF/FTC	*	*	*	*

* No hubo comparaciones con Lopinavir/Ritonavir

### Comparaciones indirectas

Se pudieron obtener resultados para realizar una comparación indirecta entre AZT/3TC vs TDF/FTC por medio del comparador común ABC/3TC cuando el tercer fármaco fue EFV, para el desenlace proporción de pacientes con carga viral <50 copias/mL a las 48 semanas. Para esta comparación no hubo comparaciones directas en este subgrupo, por lo cual la geometría de la red varía con respecto a la inicialmente planteada (Fig. 4). Esta comparación cumplió el principio de transitividad en cuanto a los factores modificadores del efecto nivel de carga viral basal y tercer medicamento. Al no poder realizar comparaciones mixtas (por ausencia de comparaciones directas luego del análisis de subgrupos, entre AZT/3TC y TDF/FTC, no se calculó el factor de inconsistencia. Aunque el metanálisis que se hizo de la comparación directa ABC/3TC vs TDF/FTC presentó alta heterogeneidad estadística (I²= 78%) mediante el análisis de subgrupos sólo se obtuvieron datos de un estudio con esta comparación directa, lo que permitió realizar las comparaciones indirectas basados en un estudio que comparó ABC/3TC con TDF/FTC y un estudio que comparó ABC/3TC vs AZT/3TC (Tabla 8). 

**Figura 4:** Red final de evidencia para el metanálisis. Los números entre los nodos equivalen a la cantidad de estudios que compararon en forma directa las intervenciones unidas por la línea. Las líneas continuas corresponden a comparaciones directas, la línea discontinuas corresponden a la comparación indirecta realizada.

**Tabla 8:** Comparación indirecta entre AZT/3TC Y TDF/FTC mediante el comparador común ABC/3TC/EFV para la proporción de pacientes con tercer fármaco EFAVIRENZ que presentan el desenlace carga viral < 50 copias/mL a las 48 semanas, diferenciados por el nivel de carga viral basal (RR - IC 95%).

	Carga viral >100,000 copias/mL	Carga viral <100,000 copias/mL
Comparaciónes directas		
ABC/3TC vs TDF/FTC	0.80 (0.64-1.00)	0.85 (0.70-1.04)
ABC/3TC vs AZT/3TC	1.00 (0.83-1.19)	1.01 (0.89 -1.15)
Comparación indirecta		
TDF/FTC vs AZT/3TC	1.25 (0.94-1.67)	1.19 (0.94-1.50)

### Evaluación de sesgos de reporte 

Con el fin de controlar los sesgos de reporte se desarrolló la búsqueda de información de acuerdo al protocolo establecido. Además se realizó inspección visual de sesgo del informe mediante gráfico de embudo para cada desenlace (se adjuntan las Figuras en el Anexo 2, observando simetría en todos, pero sin poder realizar pruebas para asimetría por el número de estudios incluidos, entre 4 a 5 en cada metanálisis realizado en la comparación directa ABC/3TC vs TDF/FTC.

## Discusión

### Resumen de la evidencia

Los resultados obtenidos en este metanálisis sugieren diferencias significativas en cuanto a los desenlaces de efectividad (proporción de pacientes con carga viral < 50 copias/mL) cuando se evaluaron comparaciones directas entre ABC/3TC vs TDF/FTC a las 96 semanas o AZT/3TC vs TDF/FTC a las 48 semanas, en favor de TDF/FTC. Sin embargo, todas las comparaciones se evaluaron mediante análisis de subgrupos, pues la revisión de las características basales en cada estudio, dejó claro que existían diferencias en cuanto al tercer medicamento utilizado, lo cual generó heterogeneidad clínica que no permitió concluir estos resultados como válidos inicialmente.

De la misma manera, reportes en estudios realizados con los medicamentos evaluados reportaron que existían diferencias en los desenlaces de acuerdo al nivel de carga viral del paciente, previo al inicio del tratamiento.

Mediante estos análisis se encontró que en las comparaciones directas de ABC/3TC vs TDF/FTC y ABC/3TC vs AZT/3TC, cuando el tercer fármaco fue EFV (con carga viral mayor y menor de 100.000 copias/mL), no hubo diferencias significativas en cuanto al desenlace de respuesta virológica a las 48 y 96 semanas. Estas comparaciones permitieron realizar una comparación indirecta entre TDF/FTC y AZT/3TC a las 48 semanas, que tampoco reportó diferencias significativas en el mismo desenlace a las 48 semanas.

De igual manera, no se encontraron diferencias significativas entre ABC/3TC y TDF/FTC cuando el tercer fármaco fue ATV/r o LPV/r. Sólo un estudio reportó un grupo expuesto a DTG como tercer fármaco sin poder obtener un comparador.

La forma de presentar los desenlaces en uno de los estudios (ABC/3TC vs TDF/FTC 3) no permitió extraer el número de pacientes expuestos y con el evento, lo cual pudo haber aumentado el total de pacientes y obtener otras conclusiones o darle mayor soporte a las ya obtenidas.

Se obtuvieron diferencias significativas en cuanto a algunos efectos adversos, específicamente supresión de médula ósea que afecta a AZT/3TC por tener mayor riesgo de presentarlo en comparación con TDF/FTC o ABC/3TC o reacciones de hipersensibilidad con ABC/3TC frente a AZT/3TC.

Los metanálisis en red se apoyan para su desarrollo adecuado en la asignación aleatoria de los ensayos clínicos que comparan las intervenciones médicas en forma directa. Lo anterior compromete un supuesto de similitud, mediante el cual sólo deben combinarse estudios si se considera que son clínica y metodológicamente similares. En los metanálisis en red las covariables que actúan como modificadoras del efecto, deben ser similares entre los estudios, la evidencia directa debe ser consistente con la evidencia indirecta y la evaluación de este supuesto debe basarse inicialmente en el juicio clínico sobre cómo afectan las diferencias la validez de este tipo de comparaciones ^30^. Lamentablemente, con los datos disponibles y la falta de cumplimiento de los supuestos de transitividad y homogeneidad, sólo fue posible realizar una comparación indirecta para un solo desenlace estudiado.

Se analizó la posibilidad de evaluar la heterogeneidad entre los estudios mediante metarregresión y análisis de sensibilidad, sin embargo, teniendo en cuenta la escasez de datos al disminuir el número de estudios potencialmente comparables de acuerdo con los factores de modificadores del efecto y a la variabilidad en las medidas resumen utilizadas en cada estudio, no fue necesario explorarla más allá del análisis de subgrupos, pues al realizarlo sólo quedó uno o ningún estudio para cada comparación. 

### Limitaciones

Si bien existe una gran cantidad de estudios que evalúan las intervenciones analizadas en esta revisión, pocos cumplieron con los criterios de elegibilidad para incluirse en este metanálisis de ensayos clínicos controlados y los mismos presentaron heterogeneidad en cuanto a la selección del tercer medicamento y en cuanto a los desenlaces estudiados o la definición de los mismos. De igual manera se encontraron potenciales sesgos en los mismos, específicamente en cuanto al cegamiento de los evaluadores o investigadores de la asignación de los pacientes.

Es importante tener en cuenta que los estudios incluidos en la revisión tienen resultados a 48 y 96 semanas, lo cual no da información más allá de este tiempo, y conocerla es fundamental para una enfermedad en la cual se pretende que la persona que la padece mantenga el control de la misma y una adecuada calidad de vida durante muchos años. 

Al mismo tiempo, los estudios reportaron sus desenlaces con tipos de mediciones que variaron entre los mismos o con definiciones que no fueron similares en algunos desenlaces, lo que limitó la extracción de datos para los análisis estadísticos. Además, se planteó un número amplio de desenlaces para evaluar la seguridad de los esquemas revisados, lo cual generó varios desenlaces negativos al no encontrarse suficiente información en los estudios incluidos y los que lo hicieron, correspondieron a subestudios con un número de pacientes menor a los que recibían la asignación aleatoria en el estudio inicial, sin poder tomar de una manera válida estos resultados pues no se podía asegurar una adecuada distribución de variables de confusión entre los grupos comparados. Se planteó a partir de esta limitación, realizar en un futuro, un nuevo metanálisis de datos individuales, con solicitud y autorización de los autores de los estudios, con el fin de estandarizar las medidas de desenlace y poder realizar obtener datos con menor heterogeneidad y poder explorar la misma mediante técnicas como la metarregresión y/o el análisis de sensibilidad. 

Otra limitación es que las guías de tratamiento actual establecen como tercer medicamento recomendado fármacos diferentes a los presentados en esta revisión y que si bien esta recomendación se basa en ensayos clínicos con asignación aleatoria, dicha asignación se realizó a partir del tercer fármaco dejando la asignación de los inhibidores de transcriptasa reversa a criterio del evaluador lo que no permitió incluirlos en esta revisión para evitar potenciales sesgos de asignación y con variables de confusión.

Por último, todos los estudios tuvieron como característica basal la realización de genotipificación previa al inicio del tratamiento, examen que permite conocer la presencia de mutaciones de resistencia a los medicamentos y con esto proveer al paciente un esquema de tratamiento con baja probabilidad de fallar por resistencia del virus. En Colombia, la guía actual no contempla la realización de este examen en este momento, sino cuando se presente la primera falla virológica, situación que limita la generalizabilidad de los resultados de los estudios analizados en nuestro país o en países donde se presente la misma situación.

## Conclusiones

Consideramos que si bien, los resultados obtenidos no muestran diferencias entre los esquemas evaluados en cuanto a desenlaces de eficacia, las limitaciones discutidas previamente no permiten que estos datos sean definitivos. Los resultados plantean la necesidad de realizar nuevos estudios que ayuden a mejorar las recomendaciones de tratamiento en los pacientes infectados por el VIH, como ensayos clínicos, con una adecuada distribución de los factores modificadores del efecto entre los grupos comparados, así como evaluar la realización de una nueva revisión sistemática con metanálisis al nivel del dato individual, con el respectivo permiso de los investigadores de los estudios incluidos o modelar los resultados en el tiempo futuro mediante técnicas estadísticas adecuadas.
